# Influence of Contact Experience and Germ Aversion on Negative Attitudes Toward Older Adults: Role of Youth Identity

**DOI:** 10.3389/fpsyg.2022.829742

**Published:** 2022-03-16

**Authors:** Yuho Shimizu, Takaaki Hashimoto, Kaori Karasawa

**Affiliations:** ^1^Graduate School of Humanities and Sociology, The University of Tokyo, Tokyo, Japan; ^2^Faculty of Sociology, Toyo University, Tokyo, Japan

**Keywords:** ageism, prejudice, contact experience, germ aversion, identity, interaction effects

## Abstract

The world’s population is currently aging, and the issue of ageism has become serious worldwide, including in Japan. Negative attitudes toward older adults can have undesirable effects on the mental and physical health of this group. We focused on the effects of contact experience with older adults and germ aversion, or the degree of aversion to infection, on negative attitudes toward older adults. Additionally, we included a moderating variable; youth identity, or the sense of belonging with younger rather than older age groups. An online survey was conducted with Japanese participants (*N* = 603). We conducted multiple regression analyses and the results showed that the interaction effect between youth identity and contact experience on negative attitudes toward older adults was significant. The findings suggest that contact experience may help in reducing negative attitudes toward older adults among people with low youth identity. The interaction effect between youth identity and germ aversion, however, was not significant. Academic research on the effects of some psychological interventions (e.g., intergenerational social exchange) should pay particular attention to the role of youth identity. Future directions for empirical studies are also discussed.

## Introduction

### Background and Purpose

The world’s population is currently aging and the percentage of the world’s population aged 65 and over is expected to rise to 17.8% in 2060 (United Nations, 2017). The issue of population aging is particularly prominent in Japan. In 2020, 28.7% of Japan’s population was aged 65 years and older (Japan Ministry of Internal Affairs and Communications, 2020), and this proportion is expected to increase. As the older population increases, intergenerational conflicts between older adults and other generations are becoming more serious worldwide (Binstock, 2010; Urick et al., 2017). Research has focused on negative attitudes toward older adults as one of the causes of such conflicts (North and Fiske, 2013, 2016). People often avoid physical and emotional proximity with older adults (Martens et al., 2005; Lee et al., 2020) and tend to have prescriptive stereotypes that older people should encourage the succession of resources, avoid active consumption of shared resources, and not assume symbolic, ingroup identity resources (North and Fiske, 2013). These negative attitudes lead to violent actions toward older adults and consequently, their poorer mental and physical health (North and Fiske, 2012; Chang et al., 2021). Thus, it is crucial to investigate the factors related to these negative attitudes that exist all over the world, including in Japan.

Our study aimed to identify the factors associated with negative attitudes toward older adults. Specifically, we focused on the effects of contact experience with older adults and germ aversion (or the degree of aversion to infection) on negative attitudes toward older adults. We also investigated a moderating variable, youth identity (the sense of belonging with younger rather than older age groups), which may affect the relationship between contact experience/germ aversion and negative attitudes toward older adults.

### Literature Review

Negative attitudes toward older adults could be related to contact experience with older adults and germ aversion. Regarding the former, people who have more frequent contact with older adults are less anxious about interacting with older adults and more likely to maintain positive attitudes toward them (Allan and Johnson, 2008). Additionally, intergenerational exchange programs between older adults and other generations are found to be effective in reducing negative attitudes toward the old (Meshel and McGlynn, 2004). Meanwhile, germ aversion could also be associated with attitudes toward older adults. People tend to avoid a person with illness-like cues regardless of whether the person has disease (Duncan and Schaller, 2009; Stevenson et al., 2011; Schaller, 2020). Older adults, especially, are often found to be cognitively linked to disease and avoided by other generations (Martens et al., 2005; Murray and Schaller, 2016; Kornadt et al., 2021; Shimizu et al., 2022). Accordingly, people with higher germ aversion are likely to have more negative attitudes toward the old.

The relationship between contact experience and negative attitudes toward older adults may be moderated by youth identity. People who have frequent contact with older people have several opportunities to experience both their favorable and unfavorable aspects (Fauziana et al., 2018; Ivan et al., 2018; Faronbi et al., 2019), and youth identity may influence which aspects are more strongly remembered. People generally have a confirmation bias, which means that they are more likely to focus on and remember the contents that match their prior image toward the target (Moskowitz and Carter, 2018; Dibbets et al., 2021). While most people will eventually belong to middle-aged and older population groups, in modern societies, more value is placed on being mentally and physically younger than one’s actual age (Cardona, 2008; Slevin, 2010; Barnhart and Peñaloza, 2013; Shimizu, 2021). Accordingly, people with high youth identity who have more contact experience with older adults may be more likely to retain the older adults’ negative aspects because they tend to consider them to be an outgroup. Conversely, people with low youth identity who have more contact experience with older adults may be more likely to remember the older adults’ positive aspects because they may not view them as outgroup members. Since previous research has focused mainly on the primary positive effects of contact experience on attitudes (Meshel and McGlynn, 2004; Allan and Johnson, 2008; Chen et al., 2017), the interaction between youth identity and contact experience on attitudes toward older adults remains unclear.

The relationship between germ aversion and negative attitudes toward older adults may also be moderated by youth identity. People with low youth identity are more likely to perceive a pathogen threat and be concerned that they may have an infectious disease. Further, those with low youth identity may have higher motivation to avoid illness and morbidity cues. Accordingly, the relationship between germ aversion and negative attitudes toward older adults may be strong in the participants with low youth identity. Conversely, people with high youth identity generally perceive themselves as healthy (Stephan et al., 2012; Messaraa et al., 2020) and may have lower motivation to avoid illness and morbidity cues. Therefore, the relationship between germ aversion and negative attitudes toward older adults may be weak in the participants with high youth identity.

Moreover, health threats increased during the time this survey was conducted due to the coronavirus disease 2019 (COVID-19) pandemic. Pathogen threats may have been felt more strongly under these conditions. Furthermore, because an early resolution to the pandemic is unlikely, and this will most likely continue affecting individuals’ daily social cognition significantly, the effects of germ aversion on attitudes toward older adults are worth investigating (Ayalon, 2020; Skipper and Rose, 2021).

### Overview and Hypothesis

Our survey focused on the relationship between contact experience/germ aversion and negative attitudes toward older adults. Especially, interactions between (1) youth identity and contact experience and (2) youth identity and germ aversion were investigated. We conducted multiple regression analyses including the above-mentioned two interactions and then a simple slope analysis.

*Hypothesis 1*: There is an interaction effect of youth identity and contact experience on negative attitudes toward older adults. Specifically, the participants with high youth identity who have more contact experience will have more negative attitudes. Whereas, the participants with low youth identity who have more contact experience will have fewer negative attitudes.

*Hypothesis 2*: There is an interaction effect of youth identity and germ aversion on the negative attitudes. Specifically, the relationship between germ aversion and negative attitudes toward older adults will be stronger in the participants with low youth identity than those with high youth identity.

## Materials and Methods

### Participants

Participants were recruited using CrowdWorks, a crowdsourcing service, and Cross Marketing Inc., a market research company. In total, 603 Japanese individuals (281 females and 322 males) participated in this study (mean age: 40.64 years, range: 18–50 years).

### Measurements

Negative attitudes toward older adults were measured using the Japanese short version of the Fraboni Scale of Ageism (Harada et al., 2004), consisting of 14 items: three items on antilocution (privately expressed prejudice; e.g., “Many old people are stingy and hoard their money and possessions”; *α* = 0.71), six on aversion/discrimination (e.g., “It is best that old people live where they would not bother anyone”; *α* = 0.77), and five on avoidance (e.g., “I personally would not want to spend much time with an old person”; *α* = 0.85). Participants rated responses on a five-point Likert scale. Mean scores of all 14 items were calculated (*α* = 0.88), and higher scores indicated more negative attitudes. The results of the analysis with each sub-concept (antilocution, aversion/discrimination, and avoidance) as the dependent variable were posted on the Open Science Framework (OSF) repository.^1^

Contact experience with older adults was measured using the question, “Do you think you have many contacts with older adults in everyday life?” The answers were rated on a seven-point Likert scale. Higher scores indicated more contact experience.

Germ aversion was measured using eight items of the Japanese version of the Perceived Vulnerability to Disease Scale (e.g., “It really bothers me when people sneeze without covering their mouths”; Fukukawa et al., 2014). Participants rated responses on a seven-point Likert scale. Mean scores were calculated (*α* = 0.77), and higher scores indicated more germ aversion. The participants’ demographic information, including gender, age, and nationality, was also collected.

Youth identity was measured using the Japanese version of the Group Identification Scale (e.g., “Would you feel good if you were described as a typical person of the youth?”; Uemura, 2001), consisting of seven items rated on a seven-point Likert scale. This scale may be used for any group or affiliation (Uemura, 2001). Mean scores were calculated (*α* = 0.84), and higher scores indicated a higher level of youth identity.

### Procedure and Analysis

An online survey was conducted, and the participants were asked to provide informed consent. On average, the survey took approximately 9 min and 3 s to complete. Data analysis was conducted using R (ver. 4.1.0). The statistical significance level was set at *p* = 0.05. All procedures performed in the study were according to the ethical standards of the ethics committee of the authors’ institution. The scale items, original data, and R scripts for analysis can be accessed *via* OSF.

## Results

### Data Screening and Summary Statistics

The participants who made a mistake when asked, “Please select ‘slightly agree’ for the item,” were considered as not having responded appropriately. Based on this criterion, 40 participants were excluded from the analysis. Data from 563 individuals (268 females and 295 males) were analyzed. The means, standard deviations (*SD*), and correlation coefficients for each variable are shown in Table 1. Consistent with the previous studies (e.g., Allan and Johnson, 2008; Shimizu et al., 2022), those who had less contact with older adults and those who had a higher level of germ aversion had more negative attitudes toward older adults.

**Table 1:** Means, standard deviations, and correlation coefficients by variable.

S. No.		*M*	*SD*	1	2	3	4
1	Negative attitudes	2.74	0.62	–			
2	Youth identity	3.15	0.97	0.03	–		
3	Contact experience	3.78	1.67	−0.22^**^	0.01	–	
4	Germ aversion	4.54	1.00	0.24^**^	−0.02	−0.10^*^	–
5	Age	40.51	7.53	−0.06	−0.29^**^	0.18^**^	0.00

*N* = 563.

* *p* < 0.05,

** *p* < 0.01.

### Hypothesis Testing

To test Hypothesis 1, a multiple regression analysis was performed on negative attitudes toward older adults involving the following variables: contact experience, germ aversion, youth identity, the interaction between youth identity and contact experience, gender, and age (Table 2). To test Hypothesis 2, another multiple regression analysis was performed on negative attitudes toward older adults involving the following variables: germ aversion, contact experience, youth identity, the interaction between youth identity and germ aversion, gender, and age (Table 3). In the multiple regression analyses, males were assigned a score of 1, whereas females were given a score of 0. The variables of contact experience, germ aversion, and youth identity were centered.

**Table 2:** Multiple regression analysis including an interaction between youth identity and contact experience.

Variables	*β*	95%CI	VIF
Youth identity	0.04	[−0.04, 0.12]	1.10
Contact experience	−0.17^**^	[−0.25, −0.09]	1.07
Germ aversion	0.25^**^	[0.17, 0.34]	1.08
Youth × contact exp.	0.12^**^	[0.05, 0.20]	1.04
Gender	0.14^**^	[0.06, 0.22]	1.08
Age	−0.04	[−0.12, 0.04]	1.15
Adjusted *R^2^*	0.12^**^	[0.07, 0.17]	–

*N* = 563. Regression coefficients are standardized. The numbers in parentheses represent 95% confidence intervals for regression coefficients and adjusted *R*^2^. Gender: Males were assigned a score of 1, whereas females were given a score of 0.

** *p* < 0.01, ^*^p < 0.05.

**Table 3:** Multiple regression analysis including an interaction between youth identity and germ aversion.

Variables	*β*	95%CI	VIF
Youth identity	0.03	[−0.05, 0.12]	1.13
Germ aversion	0.26^**^	[0.17, 0.34]	1.08
Contact experience	−0.18^**^	[−0.26, −0.10]	1.05
Youth × germ aversion	−0.03	[−0.11, 0.04]	1.04
Gender	0.14^**^	[0.06, 0.22]	1.08
Age	−0.03	[−0.11, 0.04]	1.14
Adjusted *R^2^*	0.11^**^	[0.06, 0.16]	–

*N* = 563. Regression coefficients are standardized. The numbers in parentheses represent 95% confidence intervals for regression coefficients and adjusted *R*^2^. Gender: Males were assigned a score of 1, whereas females were given a score of 0.

** *p* < 0.01, ^*^p < 0.05.

**Figure 1:**
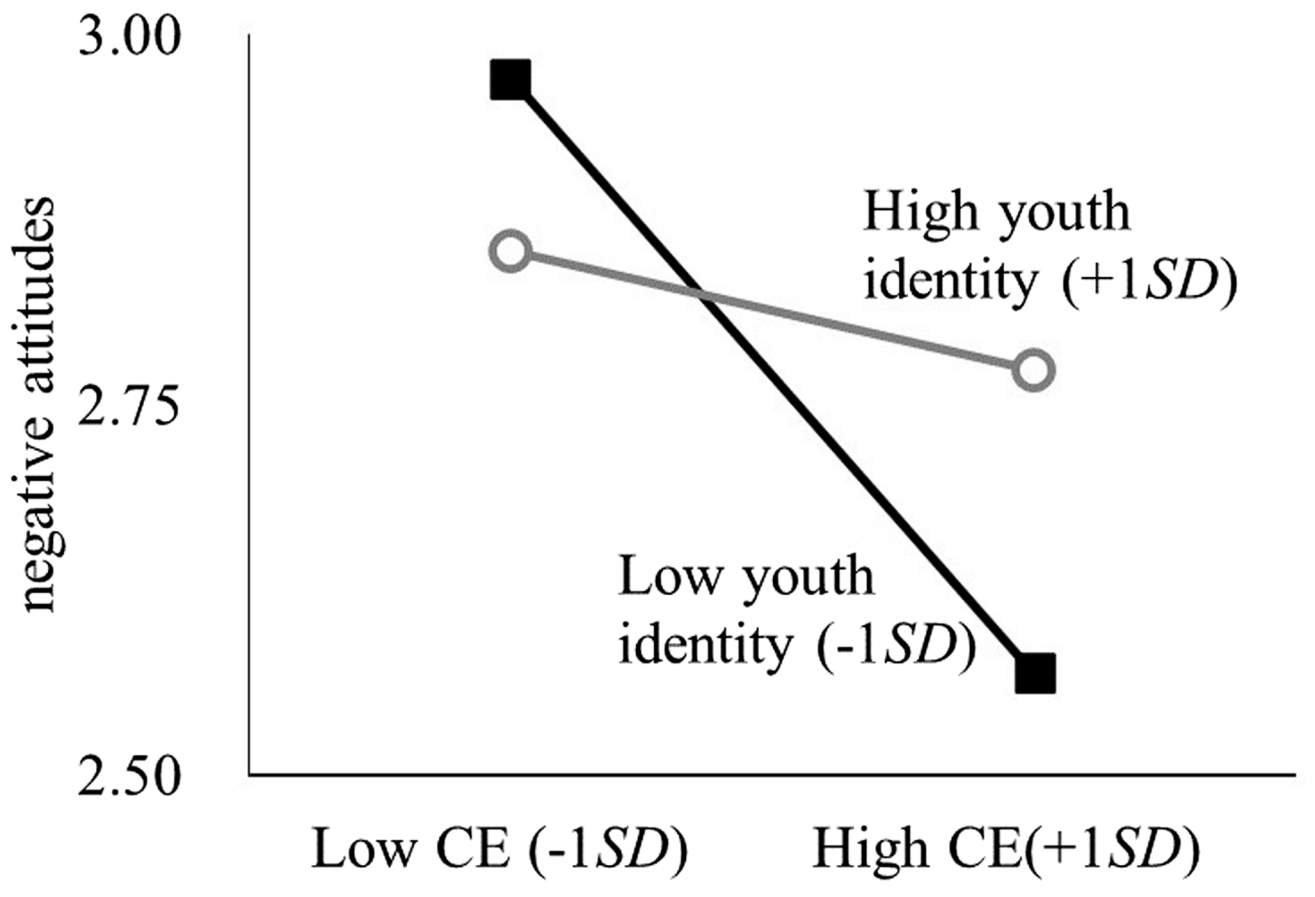
The interaction between youth identity and contact experience on negative attitudes. CE, contact experience.

The interaction between youth identity and contact experience significantly affected negative attitudes toward older adults. A simple slope analysis was conducted to examine the effect of contact experience on negative attitudes for participants with high/low (± 1*SD*) youth identity, controlling for the participants’ germ aversion, age, and gender. Contact experience had a significant negative effect on negative attitudes when youth identity was low (*β* = −0.12, 95%CI = [−0.16, −0.08], *p* < 0.001). Therefore, Hypothesis 1 was supported. Figure 1 shows the effect. Meanwhile, the interaction between youth identity and germ aversion was not significant (Table 3). Thus, Hypothesis 2 was not supported.

Further, results from another multiple regression analysis, including other interaction effects (contact experience × germ aversion, the three-way interaction), were posted on OSF. Despite this, the findings concerning the hypotheses did not differ from those presented in the main text.

## Discussion

In this study, contact experience and germ aversion as factors that relate to negative attitudes toward older people were examined. The results showed an interaction effect of youth identity and contact experience on negative attitudes toward older adults, but the interaction between youth identity and germ aversion was not significant. Additionally, men had relatively more negative attitudes toward older people, consistent with previous studies (Kalavar, 2001; Nicol et al., 2021).

### The Interactions Related to Youth Identity

The interaction between youth identity and contact experience had a significant effect on negative attitudes toward older adults. Participants would have focused on and remembered the contents that matched their prior image toward older adults (Moskowitz and Carter, 2018; Dibbets et al., 2021). Specifically, if people with low youth identity have more contact with older adults, they are more likely to see their positive aspects (e.g., warmth and kindness) because they do not consider them an outgroup (Tajfel, 1981; Fiske et al., 2002). In this study, there was a significant negative relationship between contact experience and negative attitudes toward older adults. These findings were consistent with previous studies that implemented some interventions such as intergenerational social exchange (Meshel and McGlynn, 2004; Allan and Johnson, 2008; Chen et al., 2017). However, given the results regarding the interaction between youth identity and contact experience, it is suggested that contact experience can positively reduce negative attitudes among people with low youth identity than people with high youth identity. Therefore, future research on the effects of intergenerational social exchange should pay particular attention to the impact of youth identity.

There was no significant interaction between youth identity and germ aversion. However, there was a relationship between those with a higher degree of germ aversion and more negative attitudes toward older adults. A possible reason is that this survey was conducted between August and December 2020, when people generally perceived a strong link between older adults and COVID-19 (Skipper and Rose, 2021). Regardless of the degree of youth identity, a stronger cognitive association between older adults and the virus was considered, differing from usual trends. This may have led to the lack of a significant interaction between youth identity and germ aversion, while only germ aversion had a significant main effect. Generally, older people are at greater risk for COVID-19, and it seems inevitable that citizens will associate them with the virus. However, as recent studies suggest (Ayalon, 2020), better ways to disseminate information about COVID-19, to curb the excessive reinforcement of negative attitudes toward older adults, must be explored. Furthermore, the mean for germ aversion in this study was higher than the mean (male = 3.62, female = 3.76) reported by Fukukawa et al. (2014); thus, the threat of COVID-19 may have had abnormal effects on germ aversion. Therefore, the interaction between youth identity and germ aversion when the pathogen threat is low should be re-examined.

### Limitations and Future Directions

This study has two significant limitations. First, when measuring negative attitudes toward older adults, whether participants recalled specific older people was not examined. Older adults comprise a diverse social group, and people may hold different attitudes toward various sub-categories (Brewer et al., 1981). Therefore, future research should perform detailed examinations on negative attitudes toward sub-categories of older adults. Second, contact experience with older adults was measured by a single abstract item. While the item qualified for inquiry about contact experience with older people in general, contact experience with older people who are strangers and those who are family members are considered somewhat qualitatively different. Particularly, intimate contact experience with older people in the family, such as grandparents, has been shown to be effective in affirming attitudes toward older people in general (Harwood et al., 2005; North and Fiske, 2012). Therefore, it will be necessary to further subdivide the target of contact experience and examine this in detail.

Despite these limitations, this study’s findings are meaningful for reducing negative attitudes toward older people and have implications for improving existing intervention strategies (e.g., intergenerational social exchange; Meshel and McGlynn, 2004; Allan and Johnson, 2008; Chen et al., 2017). In terms of the study findings and confirmation bias (Moskowitz and Carter, 2018; Dibbets et al., 2021), when implementing such strategies for people with high youth identity, it would be adequate to encourage them to focus on and remember the positive aspects of older adults. In future empirical studies, it may also be effective to temporarily lessen participants’ focus on age by drawing their attention to categories involving a shared identity, such as nationality when interacting with older adults.

## Data Availability Statement

The datasets presented in this study can be found in online repositories. The names of the repository/repositories and accession number(s) can be found in the article/Supplementary Material.

## Ethics Statement

The studies involving human participants were reviewed and approved by the Ethics Committee of the University of Tokyo. Written informed consent for participation was not required for this study in accordance with the national legislation and the institutional requirements.

## Author Contributions

YS, TH, and KK contributed to the conception and design of the study, manuscript revision, read, and approved the submitted version. YS organized the database, performed the statistical analysis, and wrote the first draft of the manuscript. All authors contributed to the article and approved the submitted version.

## Funding

This research has been supported by JSPS KAKENHI 16H03726.

## Conflict of Interest

The authors declare that the research was conducted in the absence of any commercial or financial relationships that could be construed as a potential conflict of interest.

## Publisher’s Note

All claims expressed in this article are solely those of the authors and do not necessarily represent those of their affiliated organizations, or those of the publisher, the editors and the reviewers. Any product that may be evaluated in this article, or claim that may be made by its manufacturer, is not guaranteed or endorsed by the publisher.

## Footnotes

^1^ https://osf.io/rce6q/?view_only=d4cd012cfa74456dac3582f00c7b5e93
